# Potential profound fluctuation in tacrolimus concentration on consumption of pomegranate rind extract: A Pharmacokinetic Experiment

**DOI:** 10.3389/fphar.2023.1140706

**Published:** 2023-04-19

**Authors:** Ritu Karwasra, Sayeed Ahmad, Surender Singh

**Affiliations:** ^1^ Department of Pharmacology, All India Institute of Medical Sciences, New Delhi, India; ^2^ Central Council for Research in Unani Medicine, Ministry of AYUSH, Government of India, New Delhi, India; ^3^ School of Pharmacognosy and Phytochemistry, Pharmaceutical Education and Research, Jamia Hamdard University, New Delhi, India

**Keywords:** rheumatoid arthritis, pomegranate, pharmacokinetics, tacrolimus, anti-inflammatory, antioxidant, CYP3A4 inhibition, LC-MS/MS

## Abstract

**Background:** Presently, varied case reports demonstrated an increase or decrease in blood concentration of diverse conventional drugs, often co-administered with edible fruits, spices, or vegetables. The overarching aim of this research is to elucidate the fluctuations in tacrolimus (TAC) blood concentration on the consumption of pomegranate rind extract (PRE).

**Methods:** A pharmacokinetic (PK) study was conducted with two groups, vis-a-vis PRE + TAC (3 mg/kg) and TAC (3 mg/kg) alone groups. An experimental study was conducted in three different manners: Single-dose (S) PRE (200 mg/kg), 7-day repetitive (7-R) PRE (200 mg/kg) dosing, and multiple (M) PRE doses (100, 200, 400, and 800 mg/kg). All the blood samples (approximately 300 μl) were drawn at different time intervals, i.e., 30 min, 1, 2, 4, 8, and 12 h after oral administration of TAC (3 mg/kg). The estimation of TAC in rat plasma was done using the hyphenated technique LC-MS/MS where the mass spectrometer used was a triple-stage quadrupole in multiple-reaction monitoring (MRM) mode.

**Results:** The findings depict that in comparison with the TAC (3 mg/kg) alone group with the 7-day repetitive (7-R) PRE (200 mg/kg) dosing, the Cmax was found to be 9.03 ± 1.21 ng/ml; AUC from time zero to infinity (AUC0-∞), 61.91 ± 17.37 ngh/ml, while the TAC (3 mg/kg) + PRE group exhibited an increase in PK parameters of TAC (Cmax 22.48 ± 3.07 ng/ml; AUC0-∞ 153.08 ± 13.24 ng h/ml). The authors further investigated in what manner the PRE affects the PK of TAC in animals. For this, docking studies with major phytoconstituents present in the PRE with CYP3A4 isoenzyme were carried out. Ellagitannins (dock score, −11.64) and punicalagin (dock score, −10.68) were again used for molecular simulation studies with TAC. To validate our findings, a CYP3A4 inhibitory *in vitro* assay was conducted.

**Conclusion:** Based on the integrated *in vivo* and *in silico* studies, we concluded that pomegranate rind extract interacts strongly with CYP isoenzyme and is therefore responsible for the altered PK profile of TAC.

## 1 Introduction

Tacrolimus (TAC) is a calcineurin inhibitor extensively used in the treatment of organ transplantation, psoriasis, and rheumatoid arthritis (RA) as a potent immunosuppressant agent (Schwartz and Mengle-Gaw, 2006; Bowman and Brennan, 2008). Several pre-clinical studies confirmed the role of TAC in the treatment of RA, and it is an approved drug for RA in Japan, Europe, and the United Kingdom (Dutta and Ahmad, 2011). TAC belongs to a narrow therapeutic window drug, and the oral pharmacokinetics (PK) of this drug showed variability in transplant patients (Mancinelli et al., 2001; Staatz and Tett, 2004). Studies revealed that TAC is a substrate for CYP3A4 and P-glycoprotein that might contribute toward its variable oral PK (5,6, and 7). Henceforth, chemical compounds, therapeutic drugs, or natural products that either inhibit or induce P-glycoprotein (P-gp) or CYP3A4 alter the PK of TAC (Hebert et al., 1999; Mai et al., 2003; Hebert et al., 2004; Xin et al., 2007). Therefore, it is imperative to consider the drug–drug or herb–drug interactions of TAC and take measures to manage the adverse or additive effects that hinder the treatment modality of patients (12, 13). Previously, numerous reports published in scientific literature warned on potential grapefruit–drug interactions, St John wart–drug interactions, and many others (Hebert et al., 2004; Xin et al., 2007; Pingili et al., 2016; Li et al., 2017), and thereby these herbs should not be consumed by patients while on therapy. This most negatively influenced those medications which are in a narrow therapeutic index.

Phytochemicals influence the PK profile of narrow therapeutic drugs more as they are composed of varied constituents and these phytoconstituents behave differently and exhibit diverse activities. Pomegranate (*Punica granatum*) family Punicaceae is one of the most common edible fruit and is often consumed with several drugs. Pomegranate has been used as a food and medicinal agent for many years in South America and Asia and is widely cultivated in arid and semiarid zones (Singh et al., 2018). The seeds of pomegranate are rich in punicic acid (65%) and some phytoestrogens. Its bark and roots are rich sources of alkaloids. Its juice and peels are good sources of glucose, fructose, and sucrose with some organic acids such as fumaric acid, malic acid, ascorbic acid, and citric acid. Additionally, amino acids (methionine, proline, and valine), tannins, polyphenols, and flavonoids are present in the peel and juice. Both are a rich source of polyphenols that indicates the pharmacological potential of pomegranate (Wu and Tian, 2017). Many scientific reports have stated that pomegranate possesses antioxidant, anti-inflammatory (Karwasra et al., 2019), anticancer, antidiabetic, antihypertensive, antifungal, nephroprotective (Karwasra et al., 2016), antimalarial, and antiulcer properties (Sreekumar et al., 2014). Several scientific publications also show that pomegranate juice and peel extracts showed interactions with diverse medications and could markedly increase/decrease the blood concentration of numerous drugs (theophylline, cyclosporine A, warfarin, carbamazepine, and TAC) in patients and rats (Hidaka et al., 2005; Khuu et al., 2013; Alanbaki et al., 2019; Alnaqeeb et al., 2019; Anlamlert and Sermsappasuk, 2020). Despite numerous potential pharmacokinetic studies, none of the studies reported on the consumption of pomegranate rind extract (PRE) with TAC altering its pharmacokinetics and the impact of long-term consumption of PRE on TAC. Second, the drug TAC lies within a narrow therapeutic window; therefore, maintaining and achieving the target through blood concentration is imperative. Henceforth, it is crucial to have a scientific study to determine whether the impact of PRE, rich in flavonoids and ellagitannins, on TAC is dose-related or not, and the probable mechanism behind this altered PK should also be explored. The current research study was conducted in an attempt to answer these questions and consequently provides valuable insights into the effect of PRE as a single-dose (S), 7-day repetitive (7-R) dose, or various dose levels (M) on the PK of TAC.

## 2 Materials and methods

### 2.1 Chemicals and reagents

The reagents and chemicals used in the research work include TAC standard (Sigma chemicals, CA, United States, purity >98%); internal standard (IS) ritonavir (Sigma-Aldrich, United States); standardized pomegranate rind extract (Natural Remedies, Bangalore, India); Cytochrome P450 3A4 (CYP3A4) inhibitor screening kit, fluorometric (BioVision Inc. Milpitas, CA, United States; catalog no K702-200); column: Eclipse XDB C-18 (100 mm*4.6 mm*3.5 μm), and HPLC-grade methanol, water, and acetonitrile (Merck, Darmstadt, Germany). Analytical grade reagents are used in the study.

### 2.2 Animals

Adult healthy 4–6 weeks old Wistar rats (150–180 g) were obtained from our institutional breeding stock. All the animals were housed in clean polypropylene cages at 25°C ± 2°C temperature with three animals per cage. This was done to acclimatize the animals before the initiation of experimentation. Water and food were provided to animals *ad libitum* throughout the study. The experimental procedure was approved and reviewed by the Institutional Animal Ethics Committee, All India Institute of Medical Sciences, New Delhi, India (Animal Ethics Approval No—772/IAEC/13). Animals were fasted overnight before the beginning of the experiment, and all the experimental practices were conducted in accordance with the Indian National Science Academy 1998, revised in 2000 as “Guidelines for proper care and use of animals in scientific research.”

### 2.3 Extraction and standardization of pomegranate rind extract

Standardized hydroalcoholic pomegranate rind extract was procured from Natural Remedies Pvt. Ltd., Bangalore, India, with batch ID PC/PG12LOT03. Natural Remedies prepared the extract by this method: Coarsely powdered dried rinds of *Punica granatum* fruits were refluxed with methanol in 1:4 at 65–70°C for the duration of 1 h. The solution was filtered after the completion of all extraction processes (Supplementary Table S1). The collective filtrate was then dried under vacuum (550 mm of Hg; NMT 70°C). Brown-colored powder having a yield 15%w/w was obtained, and the authors submitted the voucher specimen vide no SS/Pharma/014/2013 of the rind extract in the inflammation laboratory in Pharmacology Department, AIIMS, New Delhi, India. The PRE was standardized and quantified for the presence of punicalagin (Supplementary Figure S1).

### 2.4 Analysis work flow

The method for the determination of TAC by LC-MS has been validated according to the USFDA guidelines, and the same method was then applied to the pharmacokinetic study. The LC-MS work was performed at Syngenta India Limited, Corlim, Goa, India. The LC parameters were optimized to determine whether the isocratic or gradient approach was to be used. After finalizing the isocratic method for chromatographic separation, the MS parameters were finalized. The aqueous linearity of tacrolimus was then carried out by spiking the blank plasma at different concentrations of the calibration/QC stock. The sample analysis procedure was carried out by including the internal standard (ritonavir) in the mobile phase. The procedure was further modified by diluting the sample to 1 ml volume. The method was validated for the calibration curve, specificity, linearity, QC samples, lower Limit of Quantitation (LLOQ), and recovery.

#### 2.4.1 LC-MS/MS technique

The assessment of TAC in all the samples was done with the help of the LC-MS/MS method. Samples were prepared following the method stated by Shokati et al. (2015). After the preparation of samples, the supernatant was then used further for investigation. An aliquot of 10 μL was injected into the LC-MS/MS system. The column used for analysis was Eclipse XDB C-18 with 100 mm × 4.6 um × 3.5 um) with a flow rate of 1.5 ml/min (LC flow split to MS as 0.45 ml/min). The mobile phase used was a mixture of solvent A (acetonitrile) and solvent B (ammonium acetate in water) including 0.1% formic acid in a ratio of 95:05 v/v with a run time of 2 min isocratic (Supplementary Table S2. The compounds were detected by tandem mass spectrometry using electrospray ionization in the positive mode and using ion transitions m/z 821.5 -> 768.0 for TAC (standard) and m/z 721.1 -> 296.1 for ritonavir (internal standard).

#### 2.4.2 Extraction procedure

**FIGURE 1:**
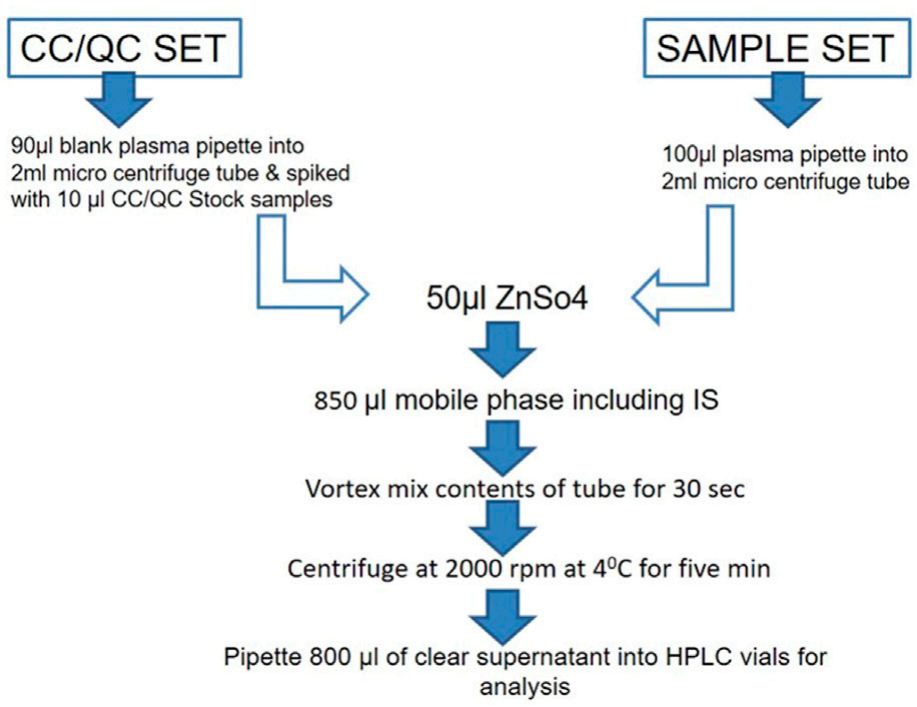
Schematic flow chart for the extraction of drug from the plasma by the precipitation method. The procedure was followed for all plasma samples, calibration curve samples, and QC samples.

Extraction of the drug from the plasma was carried out by the precipitation method. An aliquot of drug-free plasma spiked with 50 μl of ZnSO4 solution and 300 μl of methanol/acetonitrile (50:50, v/v) including IS was added. The mixture was vortex mixed for 30 s and centrifuged at 14,000 g (2000 rpm) at 40°C for 5 min. The supernatant solution was separated and filtered through a 0.45-µ membrane filter and stored at −20°C until use for analysis. The aforementioned extraction procedure was followed for all plasma samples, calibration curve samples, and QC samples. The optimization of the extraction procedure was required to reduce variation in the area of IS and improve injection to injection reproducibility. The schematic flow chart for the extraction procedure is depicted in Figure 1.

#### 2.4.3 Preparation of stock solution, calibration samples, and quality control samples

Stock solutions were prepared by dissolving an accurate amount of reference standards in HPLC-grade methanol at a concentration of 1.0 μg/ml for TAC and internal standard ritonavir. A series of working standard solutions were obtained by further diluting the stock solution in methanol. The IS working solution (200 ng/ml) was obtained by diluting the stock solution in methanol. Calibration standards were prepared by spiking the appropriate amounts of the standard solutions into 10 μL of blank plasma to yield final concentrations of 1, 3, 10, 30, 60, 100, 150, and 200 ng/ml (Supplementary Table S3). The quality control (QC) samples were similarly prepared at concentrations of 30, 800, and 1,600 ng/ml for the low-, medium-, and high-concentration QC samples, respectively. All solutions were kept refrigerated (−80°C) and brought to room temperature before use.

#### 2.4.4 Method validation of TAC

Calibration curves were constructed by plotting the peak area of the drug and concentration on the *x-* and *y*-axis separately. The accuracy and precision of linearity concentrations were calculated using the linear regression equation, y = mx + c, where x is the concentration of the drug, y is the peak area of the drug, m is the slope of the calibration curve, and c is the intercept of the calibration curve. Specificity is established by the comparison of blank plasma samples against QC samples*.* Linearity was established for 1–200 ng/ml by applying 1/x weighing factor which gave ca coefficient of variation (r2). The LLOQ of the assay is the concentration that can be measured with a defined accuracy and precision. Recovery is established by the comparison of TAC/IS ratio obtained for QC samples (LQC/MQC and HQC) against the same concentrations prepared directly in the mobile phase (FDA, 2018).

### 2.5 Pharmacokinetic experiments in Wistar rats

#### 2.5.1 Effect of single-dose p.o administration of PRE on the PK of TAC

TAC (3 mg/kg) was administered orally to experimental rats (N = 6), and in another group, PRE at a dosage of 200 mg/kg was orally co-administered with TAC dosage of 3 mg/kg in a similar manner to six rats. Both groups were given a volume of 10 ml/kg which was a single dose to all animals. Blood samples were drawn at regular intervals, and the blood plasma concentration of TAC was assessed with the help of the validated LC-MS/MS technique (Sattler et al., 1992; Alanbaki et al., 2019).

#### 2.5.2 Effect of 7-day repetitive-dose p.o administration of PRE on the PK of TAC

Pretreatment of PRE (200 mg/kg) for a period of six consecutive days was done in group 2, and afterward, on the 7th day, in group 2, PRE (200 mg/kg) was co-administered with TAC (3 mg/kg) to experimental animals (*N* = 6). In group 1, a single dose of TAC (3 mg/kg) was administered to the animals at a volume of 10 ml/kg body weight. On day 7, blood was collected from the retro-orbital sinus at regular intervals and the assessment of PK parameters of TAC was noted with and without PRE administration (Hidaka et al., 2005; Alnaqeeb et al., 2019).

#### 2.5.3 Effect of multiple doses of PRE on the PK of TAC

To study the dose–effect relationship, the Wistar rats were equally distributed into five groups (*N* = 6 rats/group). PRE at a dose of 100, 200, 400, and 800 mg/kg was given by oral route to the rats, co-administrated with TAC (3 mg/kg). The drug TAC at a dose of 3 mg/kg was given similarly to all the rats in another group, with a volume of 10 ml/kg body weight. Blood was collected from the retro-orbital plexus, and the plasma samples were separated. The concentration of TAC in the plasma samples was noted in all the aforementioned animals by the validated LC-MS/MS method (Hidaka et al., 2005).

#### 2.5.4 Blood sample collection and their treatment

**FIGURE 2:**
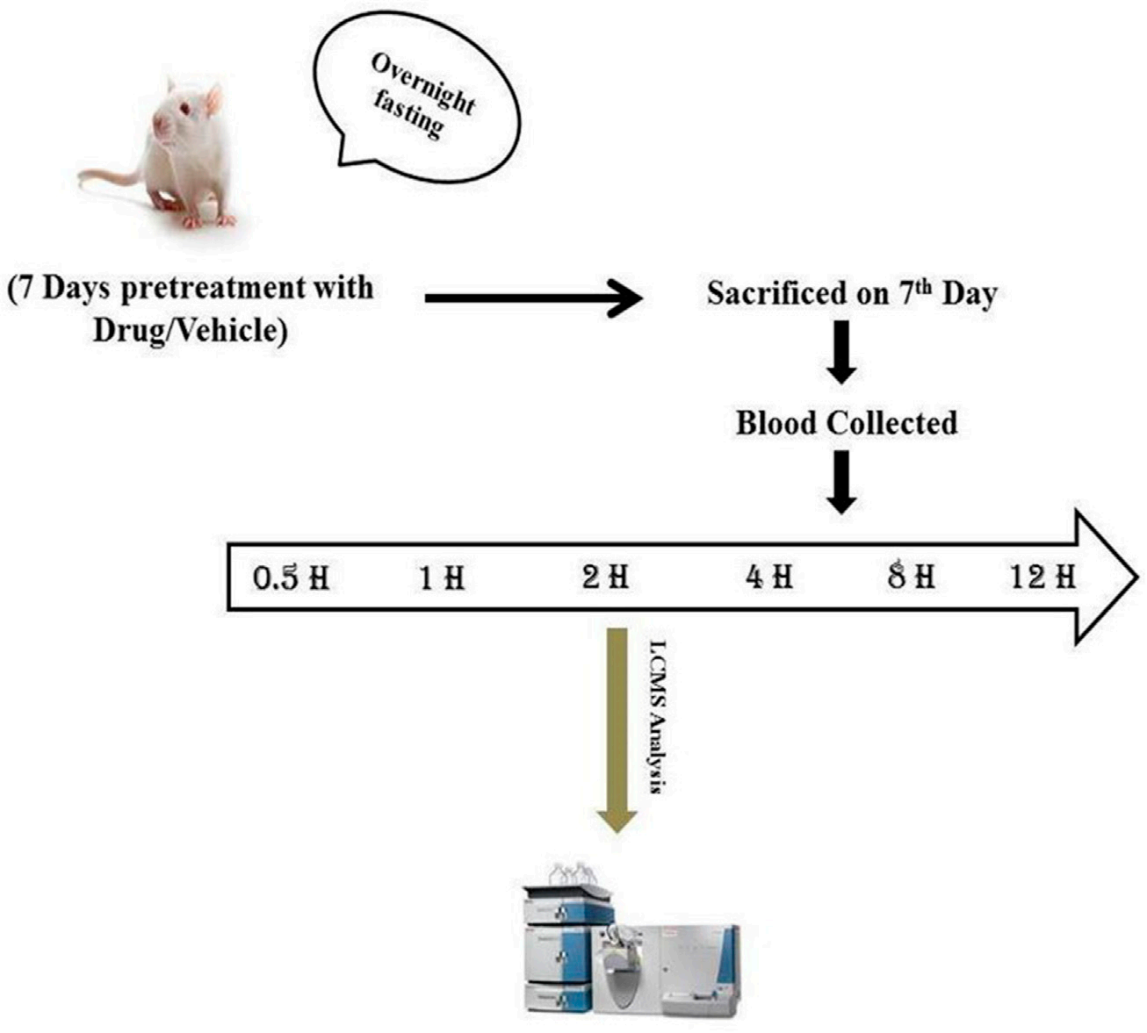
Experimental design for pharmacokinetics study. Sample blood collection at regular intervals (0, 0.5, 1, 2, 4, 8, 12, and 24 h).

The experimental animals were fasted for 12 h before the collection of blood. A total of 300 μL of blood were collected in Eppendorf tubes containing heparinized solution at different time intervals (0, 0.5, 1.0, 2.0, 4.0, 8.0, 12, and 24 h). Plasma was separated and approximately 100 μl of samples was pipetted in the tubes (Figure 2). A total of 300 μl of methanol/acetonitrile (50:50, v/v) and 50 μl of ZnSO4 solution were added, and the solution was vortexed for 30 s and centrifuged at 14,000 g (2000 rpm) for 10 min (Figure 2). All the samples were stored at −20°C until further analysis (Anlamlert and Sermsappasuk, 2020).

### 2.6 *In silico* analysis of the interaction of CYP3A4 with PRE major phytoconstituents and TAC

To verify and get clarification on our studies, we extended our studies to *in silico* analysis whereby we performed molecular docking, molecular dynamics (MD) simulation and analyzed the findings extensively. Pomegranate contains numerous phytochemicals, i.e., flavonoids, tannins, alkaloids, and phenolic compounds (ref). Since the CYP3A4 inhibitory assay of every phytochemical is cumbersome and expensive and needs a lot of resources and time, screening it with the help of *in silico* methods is a contemplative idea. These phytochemicals such as gallic acid, ellagic acid, punicalagin, punicalin, caffeic acid, ellagitannins, luteolin, kaempferol, and quercetin are interacted with protein CYP3A4 to find out their inhibitory potential.

#### 2.6.1 Molecular docking studies with CYP3A4 protein inhibitor and PRE

Molecular docking studies confirm the binding of TAC with CYP3A4 protein and also gave an idea of how the phytoconstituents of pomegranate interact with the CYP isoenzyme (Faria et al., 2007). 4D7D PDB ID (Kaur et al., 2016) was selected for CYP isoenzyme, which is bound with a native inhibitor. We removed the inhibitor, solvents, and water from it beyond 3Å for minimization. A grid file was generated around the already bound ligand, and another grid was generated on the complete protein for blind docking of all active major constituents from the PRE. A 3D structure of TAC (C44H69NO12) was downloaded from ChemSpider (393220), and for PRE, we have used multiple active constituents in 3D SDF format. All constituents were prepared and energy minimization was done. Chemical ID/ChemSpider ID for PRE constituents was provided (Table 1). After the preparation of protein and ligands, docking studies were performed using AutoDock (http://autodock.scripps.edu/) (Morris et al., 2009) and MGL tools (http://mgltools.scripps.edu/). Schrodinger’s academic maestro (https://www.schrodinger.com/freemaestro/www.deshawresearch.com) (D. E. Shaw Research, 2020) and PyMol (Rigsby and Parker, 2016) were used for analysis and visualization of the interactions.

**TABLE 1:** Active principles of pomegranate with their respective ID and databases.

S. No.	Active principle	ID	Database
1	Ellagitannin	101,601,927	PubChem
2	Punicalagin	17,216,347	ChemSpider
3	Caffeic acid	2,423	ChemSpider
4	Quercetin	5,280,343	PubChem
5	Luteolin	4,444,102	ChemSpider
6	Gallic acid	361	ChemSpider
7	Punicalin	28,428,695	ChemSpider
8	Kaempferol	4,444,395	ChemSpider
9	Ellagic acid	4,445,149	ChemSpider

#### 2.6.2 Molecular dynamics simulations with ellagitannin and punicalagin

To study the dynamic behavior of the protein–ligand complex in simulated physiological conditions, molecular dynamics (MD) simulations of the protein–ligand complex were performed using the academic version of Desmond application available with Schrodinger maestro (v 2020-4) (D. E. Shaw Research, 2020; Bowers et al., 2006). The CYP3A4-TAC complex (7764 atoms) was solvated in a 10 × 10 × 10 Å orthorhombic periodic box built with SPC water molecules. The whole system was neutralized by adding an appropriate number of 6Cl-counter ions. This solvated system was energy minimized and position restrained with OPLS3e forcefield (Jorgensen and Tirado-Rives, 1988). For CYP3A4–punicalagin (7,742 atoms) and CYP3A4–ellagitanin (7,739 atoms) complexes, 4Cl was added to neutralize the system and further solvated in a 10 × 10 × 10 Å orthorhombic periodic box built with SPC water molecules and minimized with the same OPLS3e forcefield. Ions and salt placement within 20 Å are excluded in all systems. After the system builder CYP3A4-TAC becomes 49,983 atoms, CYP3A4–punicalagin and CYP3A4–ellagitannin complexes become 49,953 and 49,989 atoms, respectively. Furthermore, 100 ns of the simulation was carried out at 1 atm pressure and 300 K temperature implementing an NPT ensemble with a recording interval of 100 ps resulting in 1,000 reading frames for each complex separately to verify and evaluate the behavior. In the end, various parameters of the MD simulation study such as the root mean square deviation (RMSD), root mean square fluctuation (RMSF), ligand binding site analysis, secondary structure element (SSE) analysis, and protein–ligand (PL) contacts were also analyzed to ensure the compactness, stability, protein–ligand interactions, and structural fluctuations in a solvated system.

### 2.7 CYP3A4 inhibitory *in vitro* assay of punicalagin

The *in silico* prediction tools gave insights into the CYP3A4 inhibition potential of phytoconstituents. A confirmatory CYP3A4 inhibition assay was conducted in accordance with the manufacturer’s instructions provided in the Cytochrome P450 3A4 (CYP3A4) inhibitor screening kit. Briefly, incubations were conducted in a reaction volume of 200 μl/well in a 96-well microliter plate. For the calibration curve, resorufin (standard) was plotted by taking different concentrations (0, 4, 8, 12, 16, 20, 30, and 40 µl) of the 1 pmol/μl of the standard available in the kit. One mole of resorufin corresponds to the metabolism of 1 mol of the CYP3A4 substrate. The reaction kinetics of no inhibitor, positive control (ketoconazole), and punicalagin standard were calculated from the rate of change in fluorescence over the time interval of no inhibitor, solvent control, and background control values. The corresponding percentage inhibition due to the test ligand or positive inhibition control was calculated. CYP3A4 activities observed with punicalagin at varying concentrations (50,100, 150, 200, 250, and 300 µM) were noted down (Hidaka et al., 2005; Faria et al., 2007). The concentration at which 50% of the CYP3A4 activity is inhibited (IC50) was noted down. The logarithm values were plotted onto the graphs. Each compound was tested in triplicates. Inhibition curves were plotted from the log values of the concentration of the samples *vs.* the percentage inhibition calculated. Ketoconazole was used as the positive control, while negative control wells contained all constituents of the reaction except the inhibitors (ketoconazole and punicalagin).

### 2.8 Pharmacokinetic calculation and statistical analysis

Pharmacokinetic parameters such as time to reach the maximum concentration (Tmax) and Cmax of TAC were read from the AUCobs (observed blood concentration *vs.* time profile). AUC0-12 was calculated with the help of a linear trapezoidal rule (Drug and Statistics (DAS) software version 2.1.1). In statistical analysis, all outcomes were stated as the mean ± S.D. The assessment of PK parameters was conducted using the standard Student’s t-test.

## 3 Results

### 3.1 Standardization of pomegranate rind extract

Pomegranate rind extract was standardized with the help of a high-performance liquid chromatography system, Shimadzu LC 2010A with an UV and PDA detector (conducted by Natural Remedies, Bangalore). The standardized extract was quantified for the presence of punicalagin by HPLC analysis (Supplementary Figure S1). The quantification of punicalagin content in PRE was found to be 11.8% w/w.

### 3.2 Optimization and validation of TAC

**FIGURE 3:**
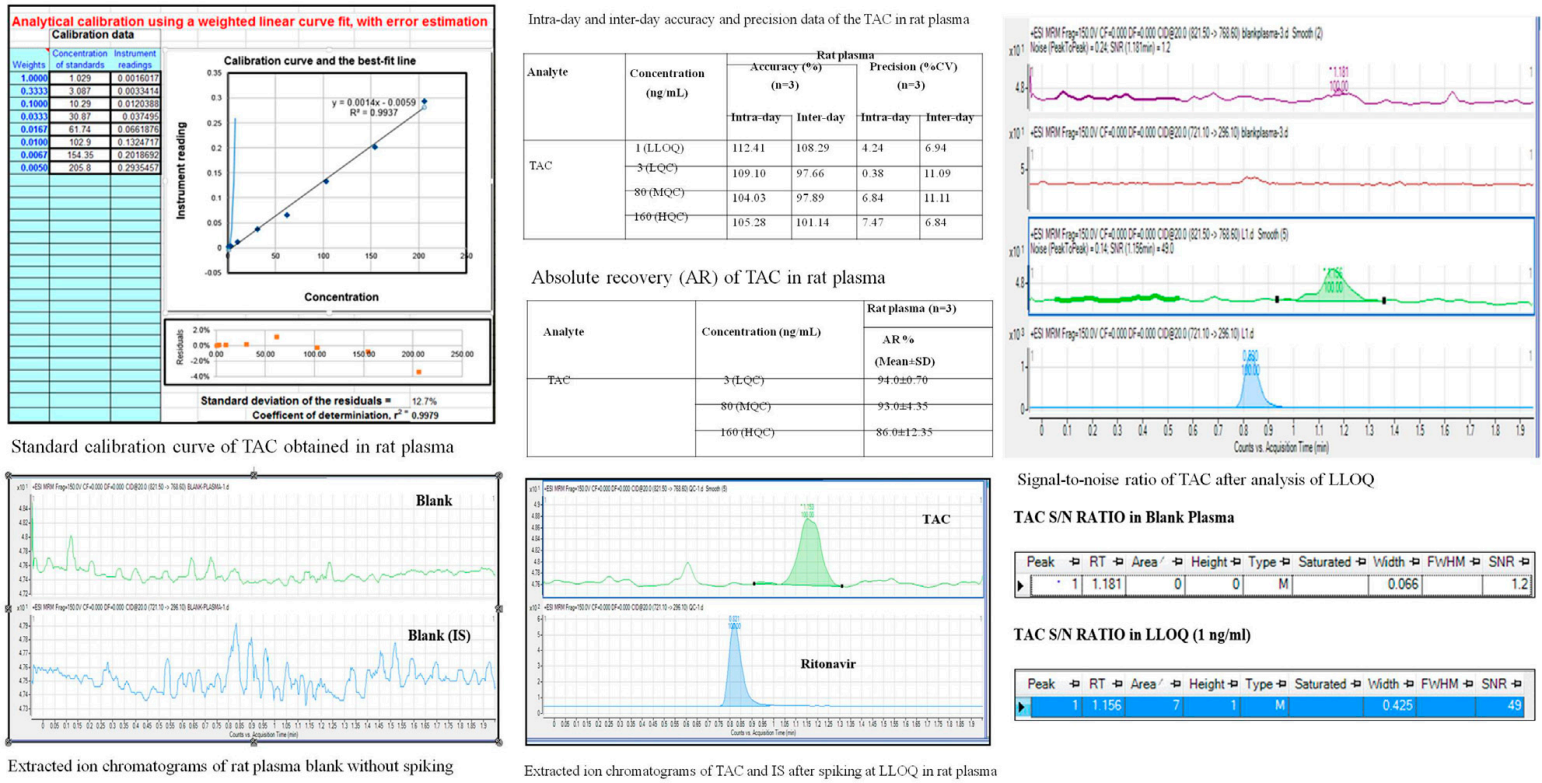
Method validation of TAC on LC-MS/MS instrument estimating QC samples, 2A; LLOQ, specificity; 2B, linearity.

The chromatographic conditions, especially the composition of the mobile phase, were optimized through several trials to achieve good resolution and symmetric peak shapes for each analyte, the IS, and a short run time. After the comparison of a few columns, the Eclipse XDB C-18 (100 mm × 4.6 um × 3.5 um) was finally selected with a flow rate of 1.5 ml/min (LC flow split to MS—0.45 ml/min) to achieve efficient chromatographic separation of the analytes and the endogenous plasma components for eliminating the matrix effects. The mobile phase consisted of the mixture of solvent A (acetonitrile) and solvent B (10 mm ammonium acetate in water) including 0.1% formic acid in the ratio of 95:05 (v/v) with a run time of 2 min isocratic (Figure 3).

#### 3.2.1 Specificity

It is established by the comparison of blank plasma samples against QC sample LLOQ 1 ng/ml. No peak is observed in blank plasma samples for TAC (1.2 min) and IS (0.8 min).

#### 3.2.2 Establishing a lower Limit of Quantitation

The LLOQ, established as a signal, observed for 1 ng/ml solution is more than 10 times the S/N ratio for blank plasma.

#### 3.2.3 Recovery

The following is established by the comparison of TAC/IS ratio obtained for QC samples (LQC/MQC and HQC) against the same concentrations prepared directly in the mobile phase: recovery at all levels was found to be more than 70%.

#### 3.2.4 Linearity and accuracy

It was established for 1–200 ng/ml by applying a 1/x weighting factor which gave a coefficient of variation (r2). At least 67% of all QC sets in one analytical sequence must pass the set limits. At least 50% of all QC samples in each level must pass the set limits. Accuracy at LLOQ lies between 80%–120% and LQC/MQC/HQC levels, below 115%.

#### 3.2.5 Aqueous linearity of TAC

Aqueous linearity of TAC was established in methanol in 1 ng/ml stock solution. Calibration standards were prepared by spiking an appropriate amount of TAC solution into methanol and analyzed by using weighted linear regression.

### 3.3 Effect of a single-dose PRE on PK of TAC

**FIGURE 4:**
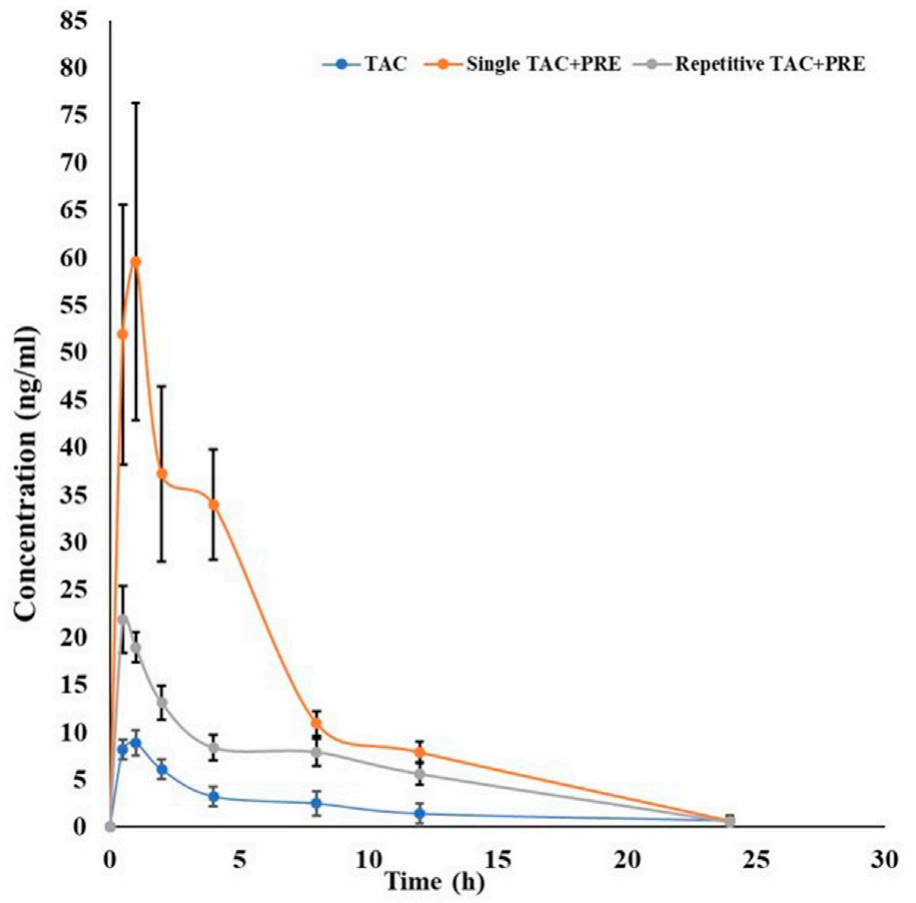
Area under plasma TAC concentration *vs.* time profile (single-dose study).

In the single-dose oral administration of the PRE with TAC (3 mg/kg) group, the mean plasma concentration of TAC was low at 0.5 h, but afterward, it tend to increase markedly as compared to the TAC (3 mg/kg) alone group. The impact of PRE administration on TAC tends to increase the mean plasma concentration of TAC. The area under the blood concentration–time curve (AUC) of PRE + TAC (*p* < 0.05) is increased with the AUC of TAC from 0 to 12 h (Figure 4). The Cmax (ng/ml) of PRE + TAC was found to be 66.27 ± 12.58, while for TAC, it is 9.03 ± 1.21. AUC 0-t (area under the plasma concentration–time curve from time zero to last sampling time) and AUC0-α (area under the plasma concentration–time curve from time zero to infinity) were increased by approximatelyfive-fold when TAC was co-administered with PRE. The apparent elimination half-life (t1/2) of TAC was longer when it was administered with PRE (0.20 ± 0.03 h), while t1/2 of TAC alone was 0.12 ± 0.04 h. Tmax was decreased in TAC + PRE single dose (0.83 ± 0.23 h) in comparison to TAC (0.91 ± 0.18 h).

### 3.4 Effect of 7 days repetitive-dose PRE on PK of TAC

PK interactions between PRE and TAC were studied *in vivo* in Wistar rats. We observed that the mean plasma concentration of TAC was increased during the administration of PRE (for seven consecutive days). In repetitive-dose PRE administration, the average plasma concentration of TAC was more than the concentration of the TAC alone group (Table 2). A different scenario in PK parameters was noted in this study. The Cmax (ng/ml) of the TAC group is 9.03 ± 1.21, increasing to 22.48 ± 3.07 (PRE + TAC group). The authors noted an increase in area under plasma concentration (AUC0-12) from 54.63 ± 14.70 to 149.18 ± 11.02 ngh/ml and the AUC0-∞ (zero to infinity) was increased from 61.91 ± 17.37 to 153.08 ± 13.24 ngh/ml. AUC increased by approximately 2.5-fold when TAC was administered with repetitive-dose PRE. The elimination half-life of TAC tends to increase from 0.12 ± 0.04 to 0.15 ± 0.03 h, as the drug TAC half-life (t1/2) increases in the blood, and therefore, the elimination decreases. Tmax decreases from 0.91 ± 0.18 to 0.58 ± 0.18 h when co-administered with PRE.

**TABLE 2:** Pharmacokinetic parameters of tacrolimus in the presence and absence of PRE in a repetitive-dose study. (Cmax, observed maximum plasma concentration; Tmax, time to reach Cmax; AUC, area under concentration–time curve; MRT, mean residence time; Vz, the volume of distribution during terminal phase; and Cl, clearance).

S. No	Parameter	Unit	TAC	TAC + PRE single-dose	TAC + PRE repetitive-dose
1	C_max_	ng/ml	9.03 ± 1.21	66.27 ± 12.58	22.48 ± 3.07
2	AUC_0-12_	ngh/ml	54.63 ± 14.70	338.81 ± 26.37	149.18 ± 11.02
3	AUC_0-inf_	ngh/ml	61.91 ± 17.37	342.45 ± 28.60	153.08 ± 13.24
4	t_1/2_	Hr	0.12 ± 0.04	0.20 ± 0.03	0.15 ± 0.03
5	T_max_	Hr	0.91 ± 0.18	0.83 ± 0.23	0.58 ± 0.18

### 3.5 Effect of multiple doses of PRE on PK of TAC

**FIGURE 5:**
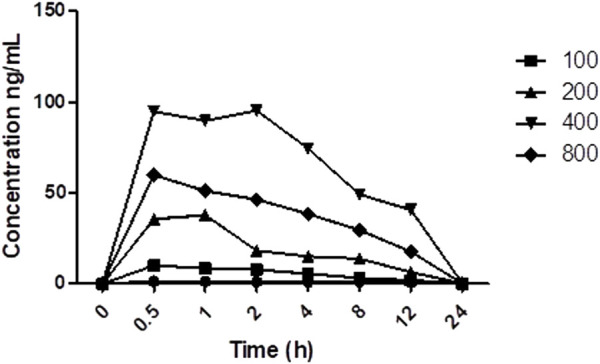
Area under plasma TAC concentration *vs.* time profile (different doses of PRE).

The average plasma concentration of TAC was altered with the co-administration of different doses of PRE (Figure 5). The average concentration of TAC increases with an increase in PRE dose, fluctuating from 100 to 400 mg/kg, but at a dose of 800 mg/kg, the mean plasma concentration does not increase (*p* < 0.01). The maximum plasma concentration was found at 400 mg/kg PRE dose, as above this dose, the concentration reaches a saturation effect. In addition, the AUC0-12 of TAC increased with an increase in PRE doses (with the same tendency). The t1/2 of TAC was prolonged when co-administered with PRE varied doses.

### 3.6 Interaction result of *in silico* analysis of PRE (active constituents) and TAC with CYP3A4

**FIGURE 6:**
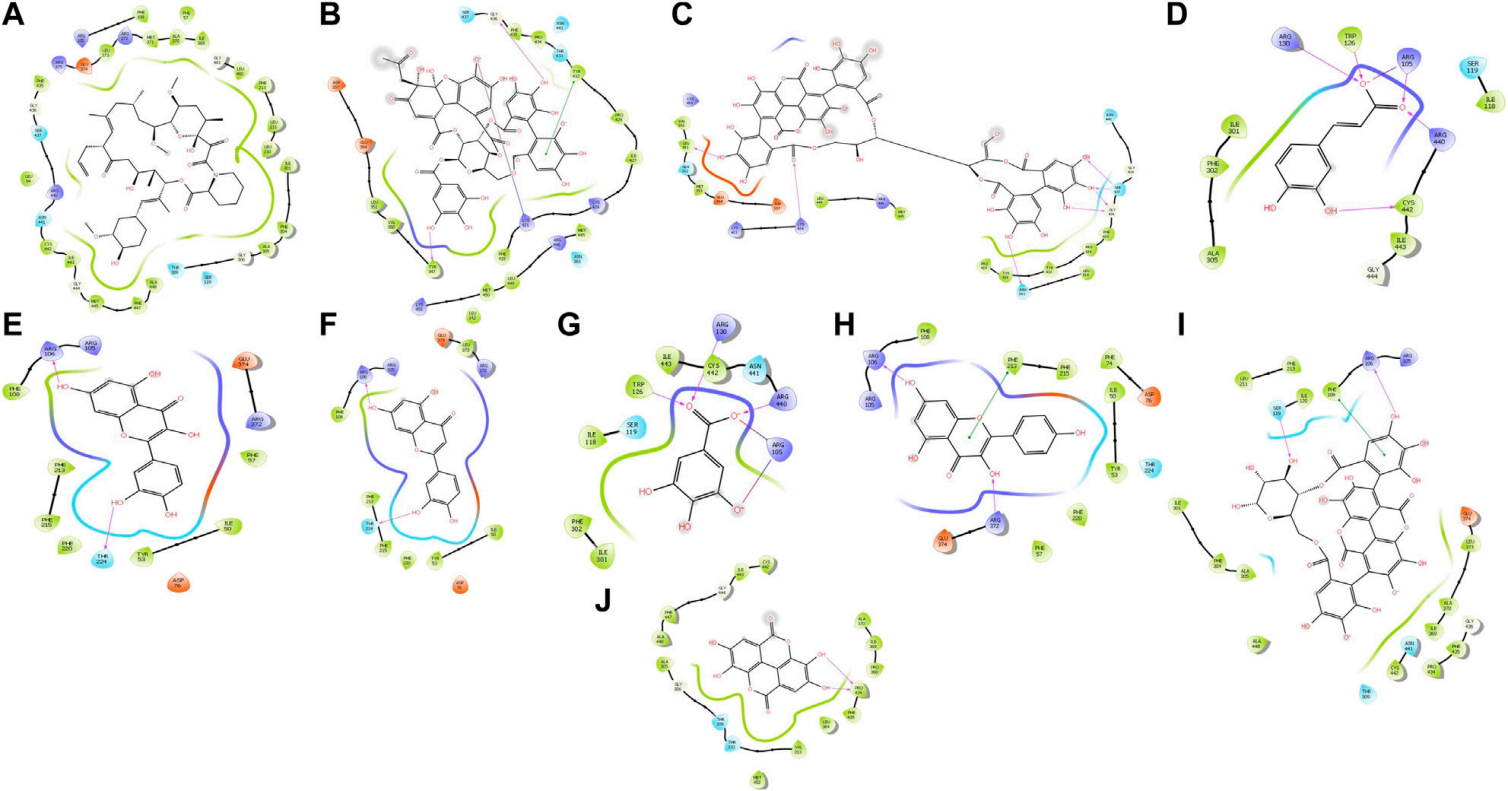
Docked ligand interaction diagram of the CYP3A4. **(A)** Tacrolimus with a docking score of −4.3, **(B)** ellagitannin with a docking score of −11.647, **(C)** punicalagin with a docking score of −10.684, **(D)** caffeic acid with a docking score of −9.593, **(E)** quercetin with a docking score of −7.971, **(F)** luteolin with a docking score of −7.105, **(G)** gallic acid with a docking score of −6.595, **(H)** punicalin with a docking score of −6.566, **(I)** kaempferol with a docking score of −6.476, and **(J)** ellagic acid with a docking score of −6.329.

Interaction with protein CYP3A4 was studied, and we found that the docking studies with CYP3A4 and TAC showed a docking score of −4.300. It was bound to the native inhibitor location of CYP3A4. Figure 6A shows that GLU374 is the only negatively charged interacting residue, while ARG440, ARG375, ARG372, and ARG105 are positively charged interacting residues. THR309, ASN441, SER119, and SER437 are polar residues with interactions. Ellagitannin shows the highest negative docking score of −11.647, which interacts with LYS421, GLY436, and TYR347 with hydrogen bond, and with TYR432, it interacts with pi–pi stacking. Punicalagin, which is known for PG main active principle, showed the docking score of −10.684 and interacts with hydrophobic residues LEU351, positively charged residues LYS424, polar residues SER437 and ASN361, and *Glycine* GLY437 with hydrogen bonds. The docking scores with their respective binding energies are presented in Table 3. The aforementioned two ligands with the highest scores, ellagitannins and punicalagin, along with the third ligand TAC were taken for MD simulation for 100 ns. Figures 6A–J show the ligand interaction representations of all active principles of PRE.

**TABLE 3:** Molecular docking score and the generated energy with respective ligands.

S. No.	Active principle	Docking score	Energy
1	Tacrolimus	−4.3	−47.072
2	Ellagitannin	−11.647	−64.541
3	Punicalagin	−10.684	−62.506
4	Caffeic acid	−9.593	−28.965
5	Quercetin	−7.971	−43.143
6	Luteolin	−7.105	−41.821
7	Gallic acid	−6.595	−28.95
8	Punicalin	−6.566	−60.616
9	Kaempferol	−6.476	−39.837
10	Ellagic acid	−6.329	−35.541

### 3.7 Molecular dynamics simulation analysis

**FIGURE 7:**
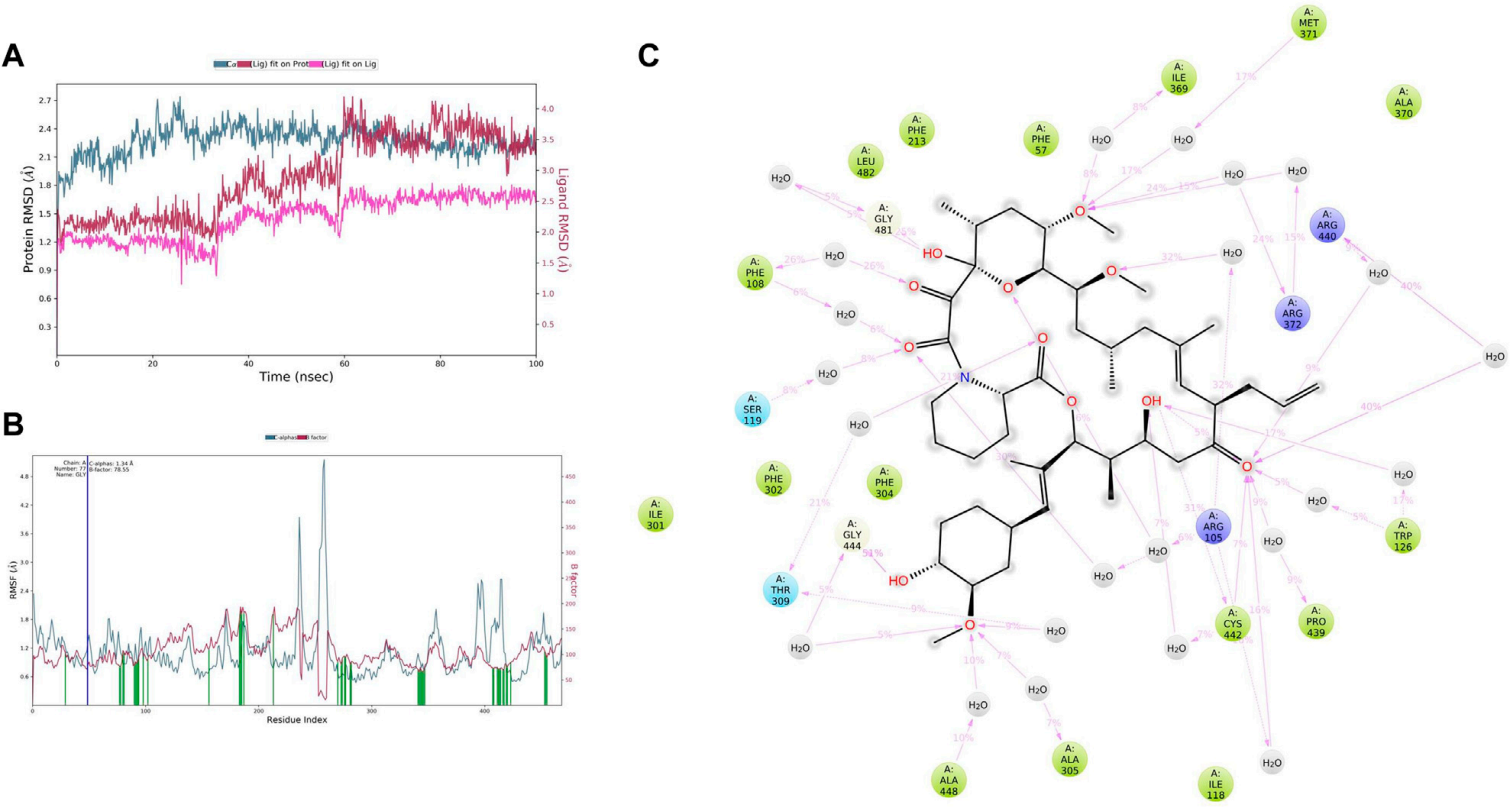
**(A)** Root mean square deviation (RMSD) of CYP3A4 and tacrolimus after the initial RMSD values were stabilized. This plot shows RMSD values for CYP3A4 on the left *Y*-axis, whereas for tacrolimus, these values are indicated on the right *Y*-axis. The RMSD graph for the c-alpha is shown in blue color, the graph for ligand fit on ligand is shown in pink color, and the graph for tacrolimus fit on CYP3A4 is shown in red color. **(B)** Root mean square fluctuation (RMSF) of CYP3A4 backbone and tacrolimus complex; red color shows the B factor, meaning the PDB, and green color means the interaction of the tacrolimus with the CYP3A4 with timescale. **(C)** CYP3A4–tacrolimus interaction during the molecular dynamics simulation.

MD simulation provides information about the receptor–ligand complex by analyzing the physical movements of atoms and molecules by allowing them to interact within a defined system and the timescale, which motivated us to perform the MD simulation for 100 ns on all three complexes generated through molecular docking. MD simulations of the protein–ligand complex were carried out using Desmond 6.1 (Maestro v12.3), and we have analyzed the trajectory files for RMSF, RMSD, and protein–ligand interactions*.* In trajectory analysis, the complex RMSD of CYP3A4*-*TAC was found within 2.12 Å, while stabilizing the structure for 100 ns of simulation. Initially, up to 50 ns, the complex’s RMSD value reached 2.4 Å and then started to decline, and we noticed that RMSD values do not fluctuate much during the complete run (Figure 7A). A total of 100 ns TAC deviated only 1.86–2.56 Å, and it was noted that ligand deviation was not much and almost constant after 5 ns during complete 100 ns dynamics. This means that the complex structure, neither protein nor ligand, has deviated much. However, the TAC fit on CYP3A4 has deviated up to 3.29 Å. The backbone atoms were observed, and the compactness, stability, protein–ligand interactions, and structural fluctuations in a solvated system were also examined. The RMSF is useful for illustrating local changes along the protein chain and is calculated throughout the simulation. It determines the flexibility of a protein region (Bowers et al., 2006). The analysis shows that the RMSF plot displays minimal fluctuations in the protein structure compared to the PDB (Figure 7B). While analyzing the C-alpha, SER286 fluctuated at 5.16 Å and GLU262 fluctuated at 1.96 Å. It was observed that the protein–ligand complex showed less flexibility, and the RMSF plot shows fluctuations in very few regions of the protein residues. For a total of 25 time periods, TAC interacted with the protein structure during the simulation. While analyzing the residue interactions during the simulation period, it was observed that the positively charged residues such as ARG372, ARG105, and ARG410 interact with water molecules, and polar residues such as THR309 and SER119 also interact with the participating water molecules. A total of 23 water molecules are involved during the simulative interactions (Figure 7C). It was also observed that hydrophobic residues such as MET371, ILE369, PHE57, PHE213, LEU108, PHE304, PHE302, ILE301, ALA418, ALA305, ILE118, CSY412, TRP126, PRO439, and ALA370 interact with the water molecules and TAC, while GLY414 and GLY481 are also found participating in the water interaction system by hydrogen bonds.

**FIGURE 8:**
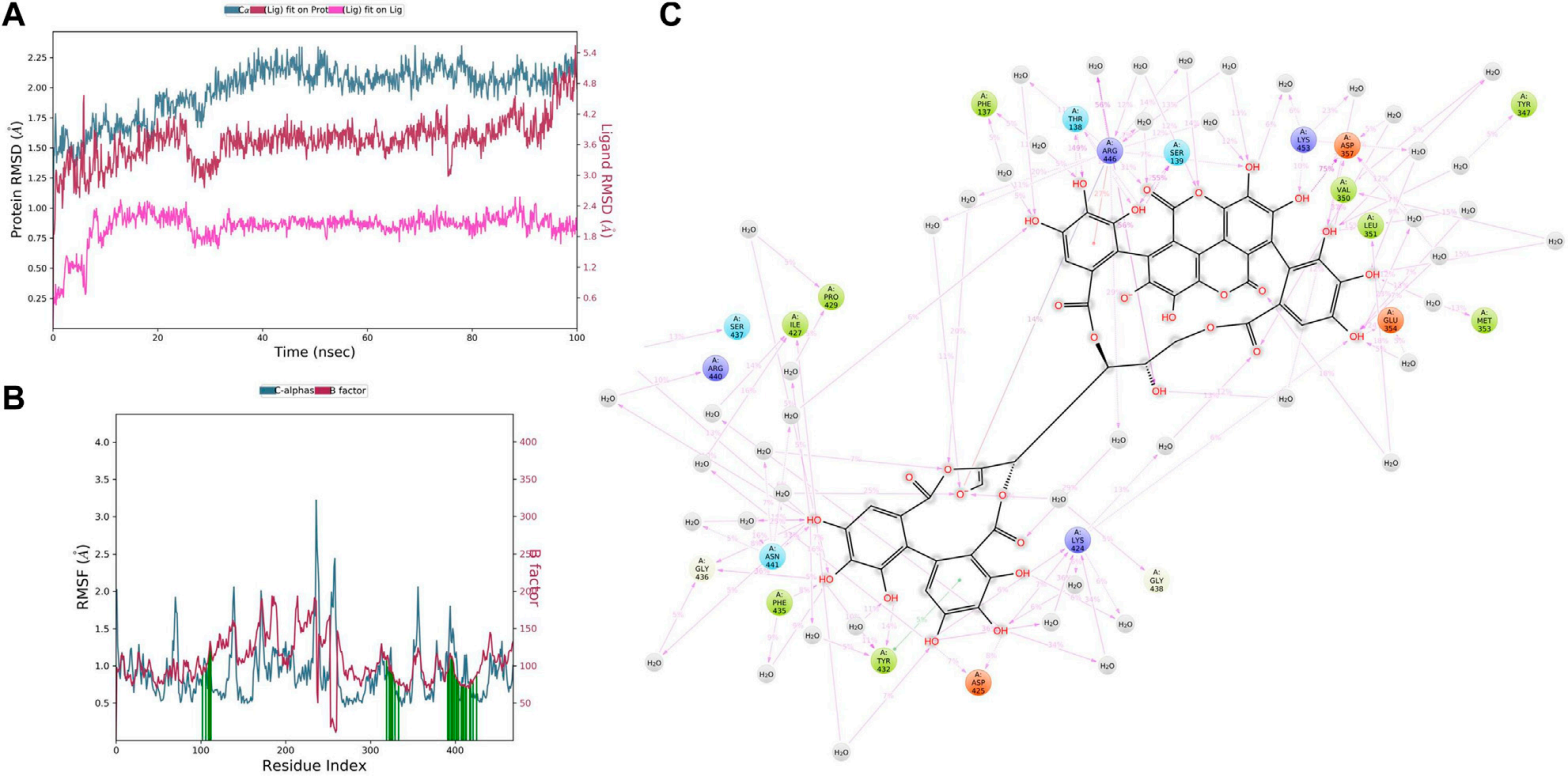
**(A)** RMSD of CYP3A4 and punicalagin after the initial RMSD values were stabilized. This plot shows RMSD values for CYP3A4 on the left *Y*-axis, whereas for punicalagin, these values are indicated on the right *Y*-axis. The RMSD graph for the c-alpha is shown in blue color, the graph for ligand fit on ligand is shown in pink color, and the graph for punicalagin fit on CYP3A4 is shown in red color. **(B)** RMSF of CYP3A4 backbone and punicalagin complex; red color shows the B factor, meaning the PDB, and green color means the interaction of punicalagin with the CYP3A4 with timescale. **(C)** CYP3A4–punicalagin interaction during the molecular dynamics simulation.

In CYP3A4–punicalagin complex trajectory analysis, RMSD was found within 2.09 Å, while stabilizing the structure for 100 ns of simulation. Initially, the RMSD of the protein was 1.44, which started to increase until 30 ns and reached 2.09 Å until 100 ns, meaning that RMSD values do not fluctuate much during the complete run (Figure 8A). A total of 100 ns punicalagin deviated from 0.61 to 2.16Å, and it is noted that ligand deviation was not much and almost constant after 10 ns during complete 100 ns dynamics. This means that the complex structure, neither protein nor ligand, has not deviated much. However, the punicalagin fit on the CYP3A4 has deviated up to 5.00Å. The analysis revealed that the RMSF plot (Figure 8B) shows minimal fluctuations in the protein structures and ends at 0.74 Å. A total of 20 time periods punicalagin interacted (green color) with the simulated protein structure. In the residue interaction diagram during the simulation period, it was observed that the positively charged residues such as ARG440, LYS424, ARG446, and LYS453 interact with water molecules and punicalagin and negatively charged residues such as ASP425, ASP357, and GLU354 interact with the OH- group of punicalagin. Polar residues such as SER437, ASN441, THR138, and SER139 also interact with the participating OH- group. A total of 48 water molecules are involved during the simulative interactions (Figure 8C). Hydrophobic residues such as PRO429, PHE435, TYR432, PHE137, ILE427, TYR347, VAL350, LEU351, and MET353 interact with water and punicalagin, while GLY438 and GLY436 also participate in the water interaction system by hydrogen bonds.

**FIGURE 9:**
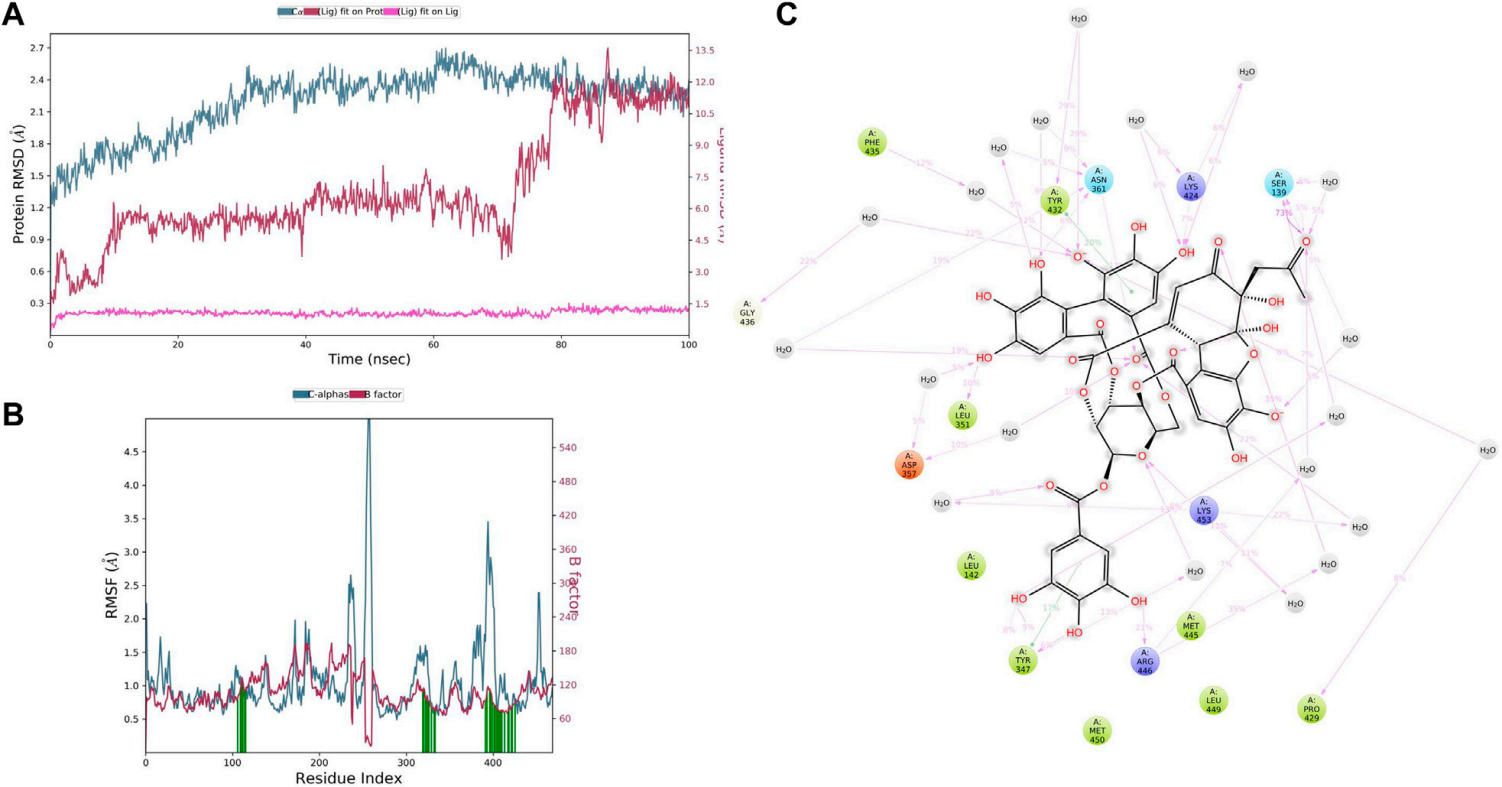
**(A)** RMSD of CYP3A4 and ellagitannin after the initial RMSD values were stabilized. This plot shows RMSD values for CYP3A4 on the left *Y*-axis, whereas for ellagitannin, these values are indicated on the right *Y*-axis. The RMSD graph for the c-alpha is shown in blue color, the graph for ligand fit on ligand is shown in pink color, and the graph for ellagitannin fit on CYP3A4 is shown in red color. **(B)** RMSF CYP3A4 backbone and ellagitannin complex; red color shows the B factor, meaning the PDB, and green color means the interaction of ellagitannin with the CYP3A4 with timescale. **(C)** CYP3A4–ellagitannin interaction during the molecular dynamics simulation.

In CYP3A4–ellagitannin complex trajectory analysis, RMSD was found within 2.34 Å, while stabilizing the structure for 100 ns of simulation. Initially, the RMSD of the protein was 1.41, which started to increase until 60 ns and went up to 2.70 Å, and then started declining until 100 ns, meaning that RMSD values do not fluctuate much during the complete run (Figure 9A). A total of 100 ns ellagitannin deviated from 1.29 to 2.30Å, and it is noted that ligand deviation was not much and almost constant after 10 ns during complete 100 ns dynamics, meaning that the complex structure, neither protein nor ligand, has not deviated much. However, the ellagitannin fit on the CYP3A4 has deviated up to 11.00 Å. In an RMSF plot, the peak indicates which region of the protein fluctuates most during the simulation, while lower RMSF values represent less conformational change. The analysis revealed that the RMSF plot (Figure 9B) displays minimal fluctuations in the protein structures and ends at 0.82 Å. A total of 20 time periods of ellagitannin interacted (green color) with the simulated protein structure. The maximum C-alpha fluctuation is seen in GLU285 with 5.65 Å. In the residue interaction representation during the simulation period, it was observed that the positively charged residues such as ARG446, LYS424, and LYS453 interact with water molecules and ellagitannin, and negatively charged residue such as ASP357 interact with the water molecules. Polar residues such as SER139 and ASN361 also interact with the participating O- of ellagitannin and water molecules. A total of 19 water molecules are involved during the simulative interactions (Figure 9C). Hydrophobic residues such as MET450, LEU449, MET445, TYR347 (pi–pi stacking), LEU142, LEU351, PHE435, TYR432 (pi–pi stacking), and PRO429 interact with the water and ellagitannin, while GLY436 also participates in the water interaction system by hydrogen bonds.

### 3.8 Effect of CYP3A4 inhibitory activity of punicalagin

**FIGURE 10:**
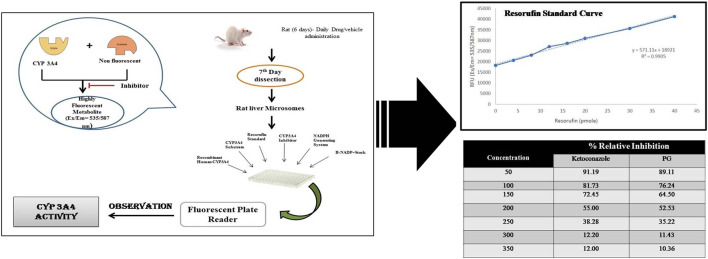
CYP3A4 percentage inhibitory activity of test compounds, positive control, and negative control.

Punicalagin at varied concentrations, i.e., 10, 20, 30, 40, 50, 60, 70, 80, 90, and 100 μg showed a different percentage of CYP3A4 inhibition. Ketoconazole was taken as positive control, and we evaluated the percentage inhibition profile of test compounds (punicalagin), positive control, and negative control (Figure 10). The authors revealed that punicalagin exhibits CYP3A4 inhibitory activity, and therefore, it can be hypothesized that the increase in blood concentration of TAC was due to this mechanism. As it inhibits the CYP3A4 isoenzyme, the metabolism of TAC was hindered. This may be the reason for the altered PK profile of TAC in the presence of PRE. Further elaborative studies are required in this assay.

## 4 Discussion

Pharmacokinetic and pharmacodynamic (PD) interactions are frequently seen in clinical practice, and their mechanistic interactions are assessed with the help of animal models. Currently, plenty of functional food/supplements/spices are often consumed with therapeutic drugs deliberately or inadvertently. Therefore, it became imperative to have scientific validation to study the impact of food supplements or edible fruits on the PK/PD profile of conventional drugs. To keep this viewpoint in mind, we initiated this research to study the impact of PRE and its phytochemicals on TAC pharmacokinetics and the mechanism behind this PK interaction.

TAC is CYP3A4 and P-gp substrate and induces metabolism in the small intestine and liver. Various drugs alter the blood concentration of TAC in patients and rats (Sattler et al., 1992; Staatz and Tett, 2004). Numerous case reports or *in vivo* animal studies have been reported on the narrow therapeutic drug, TAC. It has been reported in a study in which grapefruit juice (GFJ) inhibits the activity of P-gp, CYP3A4, and CYP3A5 in the intestine, and this interaction with TAC has been attributable to its inhibitory effect (Jorgensen and Tirado-Rives, 1988; Kaul et al., 2020; Suroowan et al., 2021). Likewise, pomelo (*Cephalocitrus grandis*) is another citrus fruit, which increased in a 2-fold concentration of TAC in renal transplant recipients (Liu et al., 2009). Another study was found in which *Schisandra sphenanthera* extract (SchE) was co-administered with TAC in healthy volunteers. They noted that the administration of SchE in healthy volunteers increases AUC, AUMC, and Cmax of TAC, whereas CL/F and V/F decrease significantly (Floren et al., 1997; Miedziaszczyk et al., 2022). TAC showed potential interactions with conventional drugs as well, and its metabolism and transport are influenced either by the induction or inhibition of P-gp or CYP isoenzymes. Ketoconazole, corticosteroids, rifampicin, sirolimus, mycophenolate mofetil, and diltiazem showed potential profound PK interactions with TAC (6,8,11). In our proposed research work, we noted the Cmax of TAC in the presence of PRE is 22.48 ± 3.07 ng/ml, and on the contrary, Cmax of TAC was measured to be 9.03 ± 1.21 ng/ml. Approximately, a 2.3-fold increase in peak plasma concentration was noted in TAC when co-administered with PRE for seven consecutive days. AUC (0–12) was noted as 22.42 ± 2.30 in the TAC (3 mg/kg) group, whereas 60.84 ± 2.85 ngh/ml was noted in the TAC + PRE group. This showed an increase in area under plasma drug concentration *vs.* the time profile of TAC. The findings corroborate to the increase in the oral bioavailability of TAC in healthy volunteers on the co-administration of ketoconazole. This increase could be explained by the ketoconazole local inhibitory effect on gut metabolism or intestinal P-gp activity (Mai et al., 2003). Studies reported so far described that the inhibition of CYP activity increases the plasma concentration of TAC, whereby the induction of CYP or P-gp activity decreases the plasma concentration of TAC. The decrease in AUC, tmax, and tmin was noted in the plasma concentration of TAC in healthy volunteers and in renal transplant patients when St John Wort co-administered with TAC (Hebert et al., 1999; Hebert et al., 2004). This alteration in the PK profile of TAC is attributable to the induction of CYP3A4 and P-gp. A variety of research reports stated that TAC showed drug–drug and herb–drug interactions, and these have become more and more common nowadays. Therefore, we investigate the impacts of a single-dose and repetitive-dose of PRE on TAC concentration. The authors found that in a single-dose administration, there is less increase in plasma TAC concentration upon the consumption of PRE. However, in the case of repetitive-dose study, there is an approximately 2.5-fold increase in Cmax and AUC (0–12). One case study published by Khuu et al. (2013) supported our study, as they noted an increase in TAC concentration when PG-containing products are inadvertently consumed by TAC transplant patients. To further explore this increase in TAC plasma concentration, we conducted PK studies with different doses of PRE and found that the increase in PRE dose increases the plasma drug concentration of TAC until 400 mg/kg but after that, no increase was noted. It signifies that CYP isoenzymes are inhibited and a further increase in CYP inhibitor concentration does not affect them.

PRE contains diverse phytoconstituents, and each phytoconstituent behaves differently (Singh et al., 2018). To elucidate which constituent is responsible for this CYP inhibitory activity, we conducted *in silico* computational studies. These *in silico* analyses when integrated with the *in vivo* findings, save a lot of time and resources. Conducting *in vivo* studies with all the phytochemicals present in the PG is cumbersome, expensive and requires a lot of resources. Therefore, this approach helps experimentalists to predict the behavior of phytochemicals or molecules in simulated environments (Egashira et al., 2012). In our study, we performed molecular docking and simulation studies. Docking analysis provides an acceptable docking score with all active principles and inhibitors. Punicalagin and ellagitannins were further selected for MD simulation studies. It gives us an idea of complex binding and significantly less deviation. TAC binds to the native ligand site of CYP3A4. Punicalagin and ellagitannin interacted with the surface of the protein with perfect docking scores. Protein RMSD was almost the same for all three ligands although the ligands RMSD varied, though all were below 1.5 Å. However, ligands fit on protein deviated but were acceptable. There were significant hydrogen bonds, hydrophobic interactions, and water bridges during the simulation period. Also, there were a good number of interactions shown by all complexes during the simulation timescale. However, ellagitannin deviated a bit but was an acceptable-range complex.

## 5 Conclusion

Based on the integrated *in vivo* and *in silico* studies, we concluded that pomegranate rind extract altered the pharmacokinetic profile of tacrolimus, an immunosuppressant drug. This herb–drug interaction could be used in both ways either as a beneficial measure (by reducing the dose of tacrolimus) or as precautionary (by avoiding the consumption of pomegranate with TAC) aspect. We conjecture that this could be used as a favorable measure to ameliorate tacrolimus-related dose-dependent side effects. Thus, there is also a need to conduct pharmacodynamics studies so that it can be used as a tacrolimus-sparing agent.

## Data Availability

The original contributions presented in the study are included in the article/supplementary materials, further inquiries can be directed to the corresponding author.

## Supplementary material

SUPPLEMENTARY TABLE S1 HPLC conditions for the quantification of punicalagin in Punica granatum extract.

Supplementary table s2 Various LC and MS parameters used for the analysis of tacrolimus.

SUPPLEMENTARY TABLE S3 Description of standard solutions, IS working solution, calibration standards, and quality control (QC) samples used for the validation of TAC in plasma.

SUPPLEMENTARY FIGURE S1 Standardization and quantification of punicalagin in PRE by HPLC analysis.

## Abbreviations

PG, pomegranate; TAC, tacrolimus; PK, pharmacokinetics; Cmax, observed maximum plasma concentration; Tmax, time to reach Cmax; AUC, area under concentration–time curve; MRT, mean residence time; Vz, volume of distribution during terminal phase; Cl, clearance; RMSD, root mean square deviation; RMSF, root mean square fluctuation; SSE, secondary structure element; PL, protein–ligand; MD, molecular dynamics; PDB, Protein Data Bank; 3D SDF, Structure data file; MGL, Molecular Graphics Laboratory.
