# Supplementary figures and images for: Potential profound fluctuation in tacrolimus concentration on consumption of pomegranate rind extract: A Pharmacokinetic Experiment
Source: Front Pharmacol. 2023 Apr 19;14:1140706. doi: 10.3389/fphar.2023.1140706 (PMC10154516; doi:10.3389/fphar.2023.1140706)

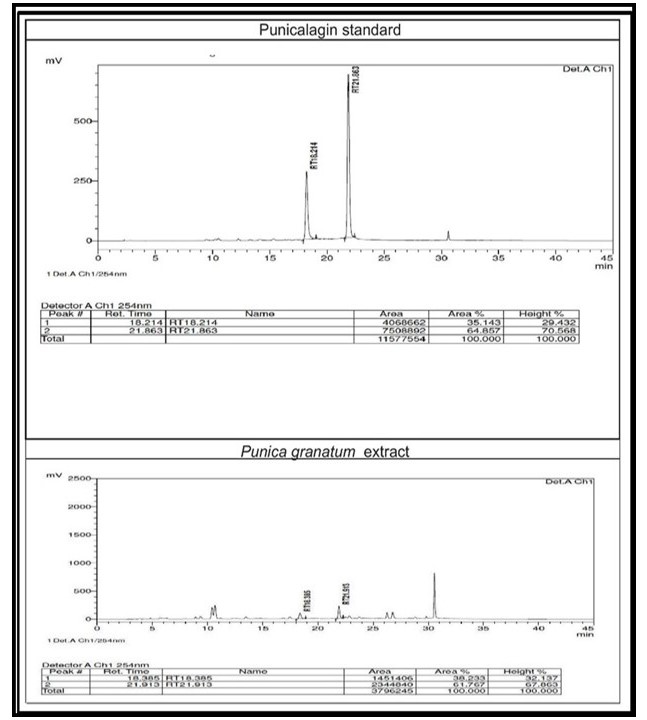

Supplement: Supplementary file 1 [file Image1.JPEG]
