# Supplementary material for: Potential profound fluctuation in tacrolimus concentration on consumption of pomegranate rind extract: A Pharmacokinetic Experiment
Source: Front Pharmacol. 2023 Apr 19;14:1140706. doi: 10.3389/fphar.2023.1140706 (PMC10154516; doi:10.3389/fphar.2023.1140706)
Supplement: Supplementary file 2 [file DataSheet1.docx]

SUPPLEMENTARY TABLE 1

| **HPLC CONDITIONS for *Punica granatum*** | | | | |
| --- | --- | --- | --- | --- |
| **Chromatographic system:** Shimadzu High Performance Liquid Chromatographic System LC2010A with UV  And PDA detector in combination with LC solution software. | | | | |
| **Chromatographic conditions** | | | | |
| Column | **:** | Phenomenex-Luna/ Hibar; 5u C-18; Size: 250x 4.60mm | | |
| Column oven temperature | **:** | 25±1oC | | |
| Mobile phase | **:** | **A –**0.140 g of anhydrous potassium dihydrogen orthophosphate (KH2PO4) dissolved in 900 ml of HPLC grade water & add 0.5 ml of orthophosphoric acid and made upto 1000 ml with water. The solution was filtered through 0.45µ membrane filter and degassed in a sonicator for 3 minutes | | |
|  | **:** | **B-**Acetonitrile | | |
| Flow rate | **:** | 1.5ml | | |
| Injection volume | **:** | 20.0µl | | |
| Gradient | **:** | Time | B. Conc (Acetonitrile) | A. conc (buffer) |
|  | **:** | 0.01 | 3 | 97 |
|  | **:** | 15.00 | 10 | 90 |
|  | **:** | 25.00 | 20 | 80 |
|  | **:** | 28.00 | 35 | 65 |
|  | **:** | 35.00 | 35 | 65 |
|  | **:** | 40.00 | 3 | 97 |
|  |  | 45.01 | Stop |  |
| Detection Wavelength | **:** | 254nm | | |
| Standard preparation |  | ~0.1mg/ml in water & injected (20µl) to HPLC | | |
| Sample preparation | **:** | ~2mg/ml in water & injected (20µl) to HPLC | | |

SUPPLEMENTARY TABLE 2

| **S.NO** | **LC PARAMETERS** | |
| --- | --- | --- |
| **1** | LC System | Agilent 1200 series quaternary LC |
| **2** | Mobile phase | 95/5 (Acetonitrile/10mm Ammonium acetate in water) including 0.1% formic acid |
| **3** | Column | Eclipse XDB C-18 (100mm*4.6mm *3.5 μm) |
| **4** | Temperature | 40°C |
| **5** | Flow rate | 1.5ml/min (LC flow split to MS - 0.45 ml/min) |
| **6** | Run Time | 2 minutes isocratic |
| **7** | Retention Time | IS (Ritonavir) - 0.8 min  Tacrolimus (TAC)-1.2 min |
| **8** | Injection Volume | 10μl |
| **S.NO** | **MS PARAMETERS** | |
| **1** | Mass Spectrometer | Agilent 6410 Triple Quad LC/MS |
| **2** | Capillary Current | 4000 V |
| **3** | Gas Temperature (N2) | 350°C |
| **4** | Gas flow | 10 l/min |
| **5** | Nebulizer | 35 psi |
| **6** | Fragmentor voltage | 150 V |
| **7** | Collision energy | 20 |
| **8** | Cell accelerator potential | 4 V |
| **9** | Ion Source | ESI (positive mode) |
| **10** | Cell accelerator potential | 4 V |
| **11** | Ms Scan Type | MRM |
| **12** | Tacrolimus | Precursor ion 821.5 768.0 m/z Product ion |
| **13** | Internal Standard | Precursor ion 721.1 296.1 m/z Product ion |

SUPPLEMENTARY TABLE 3

**Calibration and QC set**

| **S.No** | **Calibration levels (CC) (ng/ml)** | **Stock solution for spiking (10 μL) in blank plasma (ng/ml)** |
| --- | --- | --- |
| **1** | **1** | **10** |
| **2** | **3** | **30** |
| **3** | **10** | **100** |
| **4** | **30** | **300** |
| **5** | **60** | **600** |
| **6** | **100** | **1000** |
| **7** | **150** | **1500** |
| **8** | **200** | **2000** |

Calibration samples (1-200 ng/ml)

| **S.No** | **QC SET** | **Stock solution for spiking (10 μL) in**  **blank plasma (ng/ml)** |
| --- | --- | --- |
| **1** | LLOQ- 1ng/ml | **10** |
| **2** | LQC – 3 ng/ml | **30** |
| **3** | MQC- 80 ng/ml | **800** |
| **4** | HQC- 160 ng/ml | **1600** |

Quality control samples and lower limit of quantification

**Supplementary Figure 1**


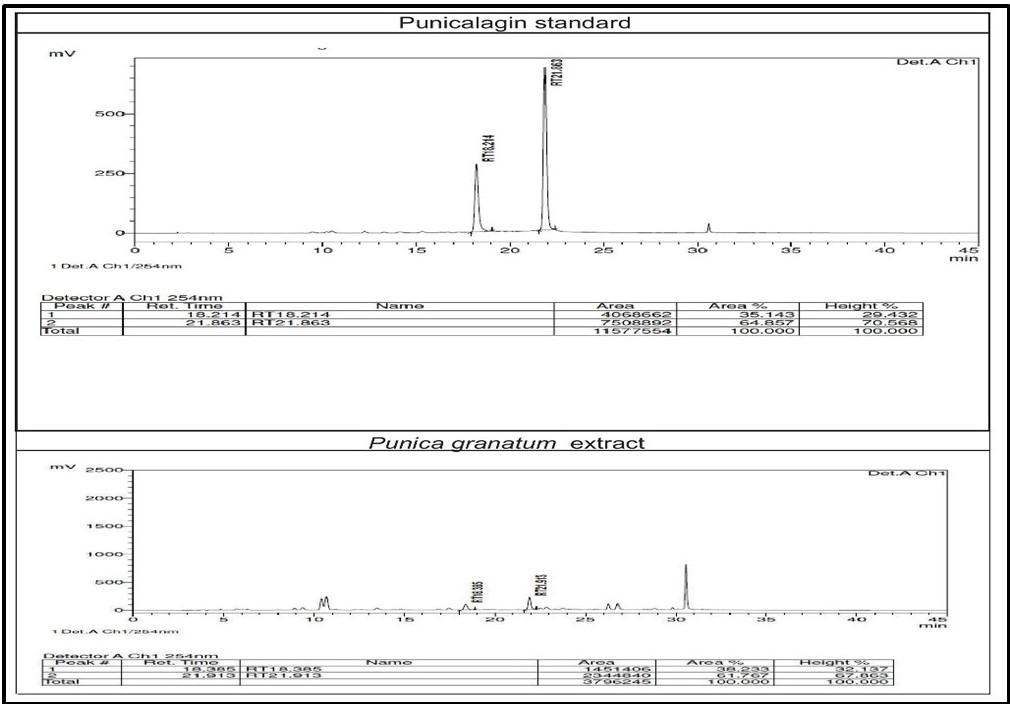


Supplementary information on standardization of PRE extract

High-performance liquid chromatography (HPLC) analysis of Punica granatum rind extract

Sample preparation: Standard concentration of 0.25 mg/ml was prepared by adding 25 mg standard punicalagin (Sigma-Aldrich, > 98% purity) to 40 ml boiled water (HPLC grade, Sartorius arium Water purification system) and sonicated for 5 min. This solution was cooled at room temperature and water was added to make volume up to 100 ml. The Punica granatum rind extract solution was prepared by dissolving 250 mg extract in 50 ml boiled water (HPLC grade), sonicated for 6 min, cooled at room temperature and water was added to make volume up to 100 ml. Both standard and sample solutions were passed through a membrane filter (diameter – 25 mm; pore size – 0.45 μm).

Instrumentation and analytical conditions

Chromatographic analysis was performed at Natural Remedies Pvt Ltd, Bangalore using reverse phase high performance liquid chromatography instrument (HPLC model - LC-2010 CHT, Shimadzu) equipped with LC10A quaternary pump and a SPD-M 10avp photodiode array detector and a UV detector adjusted at 254 nm.

Mobile phase used was composed of solution A which consisted of buffer solution, prepared by dissolving 0.140 g of potassium dihydrogen phosphate (KH2PO4) in 900 ml of water (HPLC grade), 0.5 ml orthophosphoric acid was added to adjust pH to 2.5 and water was added to make volume up to 1000 ml, and solution B consisted of acetonitrile (HPLC grade, Sigma). Components of mobile phase were filtered through membrane filter and degassed by sonification for 3 min before use. Gradient elution method was used to analyse the samples and components of mobile phase (solution A and B) were varied during the run as shown in **Supplementary table 1**. Standard or extract solution (20 μl) was injected into the HPLC system, mobile phase was run at a flow rate of 1.5 ml/min through C-18 column (250 × 4.6 mm diameter; Merck) for a run time of 45-min and separations were carried out at room temperature. Chromatographic analysis of both standard and extract was performed in triplicates, mean area and relative standard deviation were calculated using LC-Solution software.

Validation methodology

The method was validated according to International Conference on Harmonization (ICH)1996 guidelines. For the validation of HPLC procedure, parameters such as specificity, linearity, limit of detection (LOD), limit of quantification (LOQ), precision (in terms of repeatability and reproducibility), recovery and ruggedness were studied.

Specificity

Specificity of the proposed method was determined by comparing the retention time and spectra of P.granatum sample with standard punicalagin.

Linearity

To evaluate the linearity of the method, a stock solution was prepared and from this solution eight concentrations (15.5, 31.0, 62.1, 121.3, 248.6, 497.2, 994.5, 1989 μg/ml) of extract solution were prepared, which were injected five times in the HPLC system. The concentration of samples was plotted against peak area and regression equation was calculated.

Accuracy

The accuracy of the method was estimated by recovery studies using standard addition method. Pre-analyzed punicalagin samples (15.5–1989 μg/ml) were spiked with standard punicalagin (50, 100,150%) and the solution was reanalyzed by the proposed method. Percentage recovery and relative standard deviation (RSD) were estimated at each concentration level.

Precision

Precision was evaluated by determination of repeatability and reproducibility in terms of peak area and retention time. For this eight different concentrations (15.5–1989 μg/ml) were prepared, injected five times in the HPLC system and RSD was calculated.

Limit of detection (LOD) and limit of quantification (LOQ)

LOD and LOQ were determined by standard deviation (s) method from the slope (S) of calibration curve using the formula LOD = 3.3 s/S and LOQ = 10 s/S.

Ruggedness

Ruggedness was determined to assess the influence of slight alterations in the chromatographic conditions (such as change of HPLC instrument, column, changes in mobile phase composition and use of P.granatum from four different batches) on the developed method of analysis.

RESULTS

Quantification of punicalagin in *Punica granatum* rind extract using HPLC analysis

The HPLC analysis of P.granatum rind extract identified punicalagin as the active constituent present with a chromatographic peak obtained at retention time 19.2 min (Fig. 1 B). The content of punicalagin found was 11.8% w/w of dry extract.

Method validation

Specificity

The method developed was found to be very specific for punicalagin with no interference from other compounds. Peak spectra of standard punicalagin were similar to spectra of sample. The retention time for Punicalagin was found to be 19.2 min. The representative chromatograms are shown in **Supplementary Fig. 1**.

Linearity

The linearity of the calibration curve was assessed in the range of 15.5–497.25 μg/ml with a regression coefficient (r2) of 0.993 and regression equation calculated was Y = 8513.4x + 47124 (Y represents peak area and x is the concentration of punicalagin (ug/ml)) as shown

Validation parameters for HPLC method.

Parameters Range

Linearity range 15.53–497.25 μg/ml

Regression equation Y = 8513.4x + 47124

Limit of detection (LOD) 28.7 μg/ml

Limit of quantification (LOQ) 87.03 μg/ml

Precision (RSD %)

Repeatability < 2%

Reproducibility < 0.3%

Accuracy (RSD %) < 2%

Ruggedness (RSD %) < 5%

Y – peak area; x – concentration of punicalagin (ug/ml).

Limit of detection (LOD) and limit of quantification (LOQ)

LOD and LOQ calculated were 28.7 μg/ml and 87.03 μg/ml respectively.

Accuracy

The accuracy of the method was expressed in terms of percentage recovery. The accuracy of the method, as spike recovery was 95–110%, which was found to be within limit and relative standard deviation (RSD) was found < 2%. These results indicate that the developed method is accurate.

Precision

Precision of the method was expressed as RSD and it was calculated < 2% and 0.3% for repeatability and reproducibility studies respectively. These results indicate that overall reproducibility of the method developed is acceptable.

Ruggedness

Even after introducing small deliberate changes in the chromatographic method, RSD was found < 5%
